# Testing Optical Character Recognition of Home Blood Pressure Measurements, a Randomized Trial

**DOI:** 10.1093/ajh/hpaf227

**Published:** 2025-11-19

**Authors:** Austin J Strom, Esra’a Khader, Philip M Polgreen, Shelby L Francis, Alberto M Segre, Ted Herman, Lisa M Antes, Amanda R Karanikolas, Linnea A Polgreen*,

**Affiliations:** Department of Internal Medicine, University of Iowa, Iowa City, IA, United States; Department of Pharmacy Practice and Science, University of Iowa, Iowa City, IA, United States; Department of Internal Medicine, University of Iowa, Iowa City, IA, United States; Department of Internal Medicine, University of Iowa, Iowa City, IA, United States; Department of Computer Science, University of Iowa, Iowa City, IA, United States; Department of Computer Science, University of Iowa, Iowa City, IA, United States; Department of Internal Medicine, University of Iowa, Iowa City, IA, United States; Department of Internal Medicine, University of Iowa, Iowa City, IA, United States; Department of Pharmacy Practice and Science, University of Iowa, Iowa City, IA, United States

**Keywords:** blood pressure, home measurement, texting, optical character recognition, hypertension

## Abstract

**Background:**

Diagnosing hypertension typically requires repeated blood pressure (BP) readings taken over multiple days, but obtaining accurate BP measurements from patients at home is known to be challenging, at least in part due to failure to accurately report those measurements. Here, we evaluate 2 low-cost electronic reporting strategies, text messaging, and a novel image-based alternative, for acceptability and accuracy.

**Methods:**

We developed and tested a 2-stage optical character recognition (OCR) model to “read” a BP monitor display from patient-provided cellphone photographs. We then conducted a crossover trial to test the accuracy and acceptability of image-based BP reporting with having patients report BP measurements by text messaging. We compared the response rates and values obtained from photo uploads to those from text messages.

**Results:**

The majority of the 50 respondents submitted most of the requested data. We received an average of 12/14 texted BP responses and 11.3/14 photo responses from participants. Only 4 participants sent neither text nor photo responses. In addition, our OCR model achieved 98.3% accuracy when “reading” BP values from images of sufficient quality (17.9% were rejected for quality issues), and the average BP reported by subjects did not differ between our text and image-based methods.

**Conclusion:**

We were able to successfully “read” BP values from photos of BP monitors sent by participants. Both text messaging and photo uploads of monitor displays appear to be accurate and acceptable approaches for collecting home BP readings.

## Graphical abstract

**Graphical Abstract:**
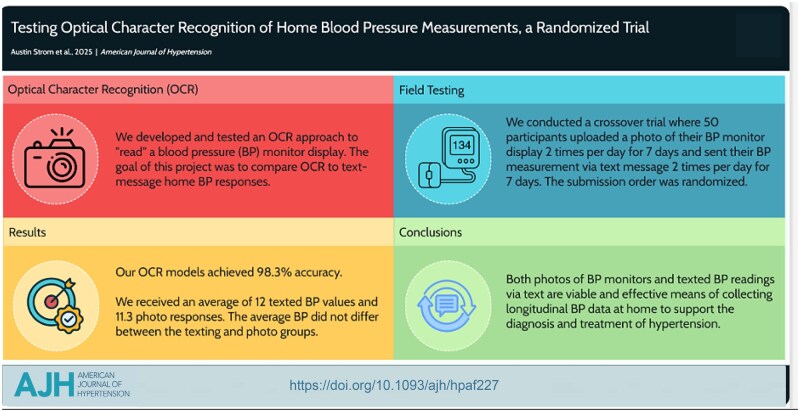

## Introduction

Hypertension accounts for a substantial proportion of the morbidity and mortality attributable to cardiovascular disease,^1^ which results in approximately 270,000 deaths per year.^2^ Hypertension is the single most important modifiable risk factor for cardiovascular disease,^3^ but diagnosing and effectively treating hypertension typically requires a series of blood pressure (BP) measurements over time.^4^ Moreover, because BP measurements tend to fluctuate, multiple measurements are also essential for ongoing monitoring of treatment effectiveness, especially after changes in therapy.^5^

Historically, most BP measurements to inform diagnosis and treatment decisions occur during clinic visits.^6^ However, many concerns have been raised about the frequency, accuracy, and reliability of in-clinic measurements due to improper technique, time pressure, or factors such as white coat hypertension.^7^ Accordingly, 24-hour ambulatory blood pressure monitoring (ABPM) has been recommended in clinical guidelines to support diagnosis and treatment of hypertension.^6^ But while ABPM is extremely accurate, the devices are expensive.^8^ As a consequence, practitioners often resort to patient-reported, non-continuous home BP monitoring using consumer-grade BP monitors instead.^6^^,^^8^

One of the limitations of using home monitoring is that measurements must be conveyed from the home to clinical decision-makers. Monitors with automatic Bluetooth or cellular reporting address this reporting problem, but can be difficult to configure, are more expensive, and often carry recurring subscription costs. Thus, many practitioners resort to either manually soliciting/collecting BP readings from patients, having patients enter their readings into their own electronic health records (EHR) via an appropriate patient interface,^9^ or having patients text their readings to their provider via short message service (SMS).^9–11^ And while these methods do not require more expensive ABPM or Bluetooth/cell-enabled monitors, they do require more work from the practitioner, may be prone to transcription/recording errors, and are difficult to validate (eg, reported values may not be recent or factual). Yet despite these shortcomings, our own prior work has shown that SMS text reporting can be a low-cost, low-effort means of collecting BP values from patients using inexpensive consumer-grade BP monitors.^10^^,^^11^

The purpose of this study is to compare the feasibility, accuracy, and acceptability of SMS BP reporting both by text and by photographic image. Patients are prompted via an SMS text message to take their BP using an inexpensive consumer-grade home monitor and then either (1) submit their BP values by return text or (2) upload a photograph of their BP monitor’s display to the link provided in the prompt, where BP values are then automatically extracted from the image. Both approaches combine the use of the patient’s own, familiar, cellphone with an inexpensive consumer-grade BP monitor: no subscriptions or additional configuration are required. The image-based approach proposed here has the added advantage of circumventing transcription errors while supporting date/time verification.

## Methods

We devised a 2-stage deep-learning-model-based optical character recognition (OCR) system. The first stage locates the BP monitor screen and its 3 measurements (systolic, diastolic, pulse) within an image, while the second stage recognizes individual digits and interprets the measurement values. Both models were based on TensorFlow Model Zoo’s 640x640 EfficientDet D1 object recognition model.^12^ To train these models, we manually constructed a corpus of 4000 BP monitor display images purposefully taken under a wide range of different conditions (eg, camera angles, lighting, background, focus, etc.). Both models were trained to reject poor quality images, such as those where digits could not be located as well as those that returned infeasible systolic/diastolic values. Post-training, our OCR system achieved 100% accuracy extracting BP values from test images of sufficient quality when measured against a human assessor. We subsequently integrated this new image-based modality into our SMS text-based BP reporting system used in our previous work.^10^^,^^11^ Further information about our OCR system is provided in the supplemental material.

### Field testing

Having established feasibility, we next performed a crossover randomized trial to test the acceptability and accuracy of our photo-based approach compared to patient-reported BP measurements sent by text message. The EHR for outpatient clinics and the emergency department of University of Iowa Health Care was scanned to identify eligible patients. Eligible participants were required to (1) be between 18 and 100 years of age; (2) have at least one BP reading above 140 mmHg systolic or 90 mmHg diastolic in the past 12 months; (3) own and use a smartphone; and (4) be able to speak and write in English. Research assistants enrolled fifty participants, who were randomly assorted into 2 equal-size cohorts based on a list of 100 random numbers. All patients were provided with an OMRON Series 3 BP monitor and were asked to take BP measurements twice a day for 2 weeks when prompted by an automated text message reminder (participants were able to choose the timing of the prompts). The first cohort was directed to upload a picture of their BP monitor display after each measurement for the first week and then report their BP measurements directly via return text message for the second week. The second cohort reported BP measurements via return text message the first week and uploaded pictures of their monitor display during the second week. At the end of 2 weeks, all participants were sent an exit survey to determine their thoughts about the study.

We recorded the following participant information: age, race, ethnicity, sex, and systolic and diastolic BP. We calculated participation, completion, and response rates for both photo- and text-based BP messaging and compared these rates using *t*-tests. All statistical analyses were performed using SAS version 9.4, and this study was approved by the University of Iowa Institutional Review Board.

## Results

**Figure 1.**
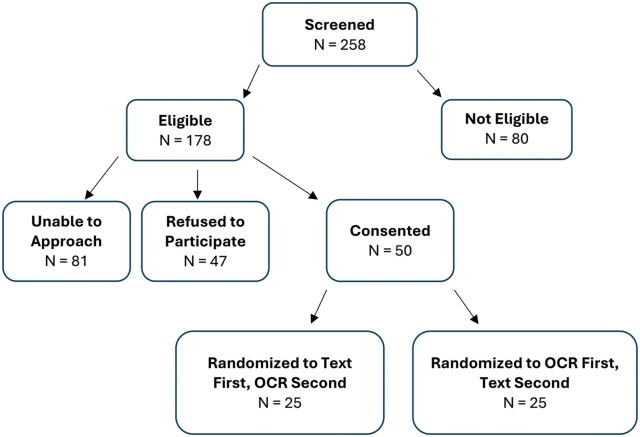
Flowchart of patient participation and follow-up throughout the study period.

There were 25 patients in the text-messaging-first group and 25 patients in the image-first group (see Figure 1). Summary statistics are shown in Table 1: the mean age of participants was 61.6 years; 52% were male; and 84% were white.

**Table 1.** Baseline characteristics for respondents who completed the study.

Baseline	Frequency (%)
**Age**	
**Less than 45 years**	7 (14%)
**45-65 years**	20 (40%)
**Greater than 65**	23 (46%)
**Race**	
**White**	42 (84%)
**African American**	7 (14%)
**Other**	1 (2%)
**Ethnicity**	
**Non-Hispanic**	49 (98%)
**Hispanic**	1 (2%)
**Sex**	
**Male**	26 (52%)
**Female**	23 (46%)
**Other**	1 (2%)
**Screening systolic BP readings**	
**120-139**	2 (4.0%)
**140-149**	24 (48.0%)
**150-159**	14 (28.0%)
**160 and above**	10 (20.0%)
**Screening diastolic BP readings**	
**Less than 80**	20 (40.0%)
**80-89**	17 (24.0%)
**90-99**	11 (22.0%)
**100 and above**	2 (4.0%)

Table 2 shows the proportion of patients who returned BP readings either by text message or by photo upload. There were no statistically significant differences between response rates (the number of text messages sent or photos uploaded): the average text response rate was 12/14, and the average photo response rate was 11.3/14 (*P* = .193). Twenty-one participants (42%) had 100% completion rates (sent the maximum number of either text messages or photos; *P* = 1.000). Six participants did not text any BPs (12%), and 7 participants did not send any photos (14%) (*P* = .767). Only 4 participants sent no text messages and no photos (8%). There were also no significant differences between participants who started with text messages and ended with photos or started with photos and ended with text messages.

**Table 2.** Average number of BP readings sent by both groups by text message or photo upload.

Group	Average number of readings	*P*-value	Number (%) who sent zero readings	*P*-value	Number (%) who sent 14 readings (max)	*P*-value
**Text-messages**	12	.193	6 (12.0%)	0.7673	21 (42.0%)	1.00
**Photos**	11.3		7 (14.0%)		21 (42.0%)	
**Text-messages (first)**	12.2	.662	3 (12.0%)	1.00	9 (36.0%)	.395
**Text messages (second)**	11.8		3 (12.0%)		12 (48.0%)	
**Photo (first)**	12.1	.182	2 (8.0%)	0.389	12 (48.0%)	.395
**Photo (second)**	10.4		5 (16.0%)		9 (36.0%)	

Averages are also stratified by whether the participants sent messages via text first, or via photo upload first.

The mean systolic BP reading was 135.2 mmHg for text messages compared to 137.4 mmHg for photo uploads (*P* = .262). The mean diastolic BP received was 80.4 mmHg for text messages and 80.0 mmHg for photo uploads (*P* = .993). These results were not statistically significantly different. As might be expected in this population, most BP measures submitted were high: 30 (65.2%) of systolic BP measurements submitted by text message were 130 mmHg or greater, and 26 (60.5%) of systolic BP measurements submitted by photo upload were 130 mmHg or greater.

**Figure 2.**
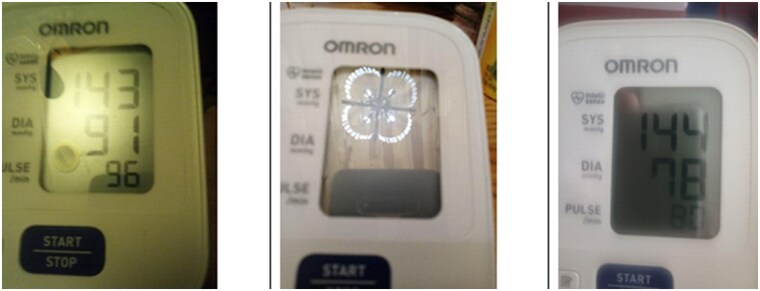
Images of BP monitor displays unreadable by our method.

We received 510 photographs via the link provided in the SMS text prompt. Six files could not be opened, and our system rejected 17.86% of the photographs uploaded. Rejections were generally caused by images with higher levels of blur, glare, or shadows (see Figure 2), although 2 were rejected because they simply had no discernible BP monitor display, possibly due to operator error (eg, failure to upload the correct photograph). Our automated OCR system achieved 98.3% accuracy (7 errors) extracting BP values from well-formed images as measured against a human assessor.

A link to an exit survey was sent to all participants upon completing the 14-day study. When asked about the difficulty of both text messaging and photo upload, participants rated the text-messaging method a little bit easier to use than the photo-upload method (4.46 vs 4.12, respectively, *P*-value = .044). These questions were on a 5-item scale, from extremely difficult (1) to extremely easy (5). Open-ended responses about texting included, “The overall process was very simple, no problem,” and about photo upload, “Wasn’t as easy to send a picture at first but it got easier.”

## Discussion

In this study, we found that most patients were willing and able to return their own BP measurements by either return text message or by photo upload upon receiving a text message reminder from our research team. We received an average of 12 texted BP responses and 11.3 photo responses over a total of 14 reminders. In addition, we found that using our 2-stage deep-learning OCR system, we were able to successfully “read” nearly all of the well-formed images submitted by participants. Finally, the average BP reported by subjects did not differ between our texting or OCR methods, and most participants said that returning BP measurements via text and OCR was either easy or very easy.

BP measurements are recorded in the EHR at most clinic visits. In recent years, EHRs have spread to most health systems, providing a convenient platform for collecting and reviewing BP measurements over time. These longitudinal measurements should facilitate hypertension diagnosis and treatment.^13^^,^^14^ However, the introduction of EHRs has not been associated with increases in hypertension diagnosis and control^15^ at a population level. In fact, as EHR use has expanded, BP rates of diagnosis and control have actually declined.^16^ Elevated BP measurements frequently fail to translate into clinical action; both diagnostic inertia and clinical inertia^17^ are common for hypertension. In many cases, elevated BP measurements are not discussed in clinical notes, even in primary care settings.^18^ Clearly, new approaches for diagnosing and treating elevated BP are needed.

The availability of accurate and inexpensive consumer-grade automated BP monitors makes non-continuous home-based measurement of BP increasingly feasible. Our results suggest 2 effective and acceptable approaches for collecting home BP measurements via SMS: the traditional text-based approach and the image-based approach described here. We believe these approaches are effective for 2 major reasons. First, both of our approaches use text prompts to remind patients to take their BP at specific times. These specific reminders are more effective than just recommending that patients take and record their BPs at home between clinic visits.^10^ The second reason we think that both of our approaches are effective relates to their use of technology (ie, texting and taking photos with their cellphones) familiar to patients in their everyday lives: according to the Pew Research Center, 98% of Americans own a cellphone as of 2024.^19^ Both approaches rely on the patient’s own, presumably familiar cellphone, and neither requires a special app, complicated pairing of BP monitors with personal devices, use of home networking infrastructure, or ongoing subscription costs. Accordingly, both of our approaches present an inexpensive, effective, and operational way to collect home BP measurements.

While texting is inexpensive, easy, convenient, and not substantially different in terms of effectiveness compared to photo uploading and OCR analysis in our study, there are some specific benefits to using OCR. First, our approach using photos helps ensure that actual BP values are transmitted: it eliminates the possibility of transcription errors or the submission of fabricated or recycled BP values. Indeed, previous research has shown that some patients might report their BP erroneously or intentionally under-report it. For example, in a study using BP monitors that saved BP readings, 50% of the patients reported results that differed from the stored values, and patients reported BP readings that were not stored in the monitor while failing to report others that were.^20^^,^^21^ These potential problems are partially mitigated by the metadata embedded in photos, which can, for example, be used to establish the date and time photos were taken. Thus, we would be able to determine if patients were submitting photos of BP values taken on days that differed from the submission date. Second, although some participants commented that the first few photo uploads were challenging, most quickly became adept, and there were no significant differences between the number of BP values received via text and the number received via photo upload.

Both text message responses and our image-based approach have some advantages over the use of Bluetooth-enabled monitoring systems. First, neither requires installing an app or linking devices to home Wi-Fi. Second, no subscription service is required: Bluetooth or cellular-enabled BP monitors that are easy to use are sometimes paired with expensive monthly subscription costs, even if, in most cases, only a relatively small collection of measurements may be needed for diagnosis or, later, after the occasional change in prescription. Third, entering BP values as text or taking a photo may help patients think more about their actual values than having values silently transmitted via a third-party service, which may as a result help make patients more aware of the importance of both making a diagnosis and controlling their BP levels after diagnosis. Finally, both methods (text-based and image-based) can be easily adapted for any BP monitor with a standard display; more expensive monitors such as Bluetooth-enabled or cellular-enabled are not needed. This work demonstrates that text and image-based responses are equally viable, effective, and inexpensive means to effectively collect longitudinal BP values from patients between office visits and after medication changes.

There are some disadvantages of texting and OCR. Specifically, with OCR, even though we can determine when the photo submitted was taken, participants could refrain from submitting some photos that show less-favorable measurements. Also, texting and OCR, unlike some Bluetooth and cellular systems, are not automatically linked to the EHR. An extra step to enter submitted BP measurements is most likely needed. The advantages and disadvantages of all 4 types of home blood pressure reporting are summarized in Table 3.

**Table 3.** Home blood pressure return methods: advantages and disadvantages.

Method	Advantages	Disadvantages
**Texting**	Quick and easy for patients Widely accessible No equipment needed beyond a cellphone Requires patient to see BP value	Data security Possible typographical errors, omission of measurements Limited EMR integration
**Bluetooth**	Automatic data transfer Eliminates transcription errors Potential for data integration with EMR	Requires compatible devices Setup can be difficult Potential extra costs (eg, subscriptions) Costly monitors
**Cellular**	Real-time remote monitoring No need for Wi-Fi or apps	Very costly monitors Device dependent
**OCR**	Easy for patients Eliminates transcription errors Only needs a cellphone with a camera Patient more likely to see BP value	Limited EMR integration Possible omission of measurements

Our study does have some limitations. First, it was performed in a single health system and may not be generalizable to other settings and populations. Second, although all participants had an elevated BP measurement recorded in the 12 months before the study, some participants had normal or near-normal BP measurements during the study period. Recruiting participants with more elevated BP levels might have led to a greater level of participation. Selection bias may have also contributed to our high success rate: many of those approached declined participation, and specifically, those who were not interested in measuring their BP might have elected to not participate. Third, readings from both image-based and text-based BP responses cannot generally be committed directly to the EHR, although the impediments to doing so are grounded more in policy than in any fundamental technical issue. Finally, although the accuracy of our OCR classifier was remarkably high on photos deemed of sufficient quality, approximately 17% of our photos were rejected for being of low quality. A manual review of these photos showed that these rejections were related to poor photo quality, which could be improved with specific instructions about lighting and technique (avoiding glare and making sure all of the numerical values are visible). In this study, no guidance was provided regarding photo quality, thus, the observed rate of rejection represents a lower bound. Future participants should be provided instruction on how to take better photos. Moreover, rapid advances in AI technology ensure that newer general purpose AI models (eg, ChatGPT 4-turbo), unlike the OCR model used here, are able to enhance and accurately extract BP readings from all but the center example in Figure 2 without the need of any additional preparation or specialized training, ensuring that updated versions of our OCR system will enjoy much lower image rejection rates as well as improvements in performance and accuracy on less-than-perfect photos.

Despite the limitations associated with this pilot study, we were able to successfully “read” BP values from photos of BP monitors sent by participants. This demonstrated that both photos of BP monitors and patient-provided BP readings via text are viable and effective means of collecting accurate longitudinal BP data to support the diagnosis and treatment of hypertension. Future work will not only involve incorporating newer, more capable AI image analysis models, but will also involve testing whether patients prefer sending images over entering texts, and Bluetooth- and cellular-enabled monitors. If successful, OCR will be used in future interventions to improve BP control.

## Supplementary Material

hpaf227_Supplementary_Data

## Data Availability

The data underlying this article will be shared on reasonable request to the corresponding author.
