# Supplementary material for: Testing Optical Character Recognition of Home Blood Pressure Measurements, a Randomized Trial
Source: Am J Hypertens. 2025 Nov 19;39(6):744–9. doi: 10.1093/ajh/hpaf227 (PMC13101530; doi:10.1093/ajh/hpaf227)
Supplement: hpaf227_Supplementary_Data [file hpaf227_supplementary_data.docx]

**Appendix**

Initial Data and Preprocessing

First, we took our own images of BP-monitor displays with varying lighting and background conditions. These images were then annotated using bounding boxes according to the 3 different measurements present in the image: systolic BP, diastolic BP, and pulse. An example of this annotation can be seen in **Appendix Figure 1**. These images were then augmented, along with their bounding boxes, using a random set of methods to cover different scenarios that the model might see. Following the first set of annotations, images were cropped based on the original bounding boxes to only include the digits present in those measurements. After cropping, these images were then annotated to classify the digits that were present in each of the measurements. In the end, there were 233 images used to train the stage one model to detect the measures which consisted of 209 images to update the weights and 24 images to evaluate the performance. Additionally, there were 4482 images to train the stage two model to detect and classify the digits in the measurements which consisted of 4033 images to train the model and 449 to evaluate its performance.

**Appendix Figure 1: Detection and identification of digits on BP monitor display.** Taking a photo of the BP display (left), reading values from the photo (center), data recorded (right)

**
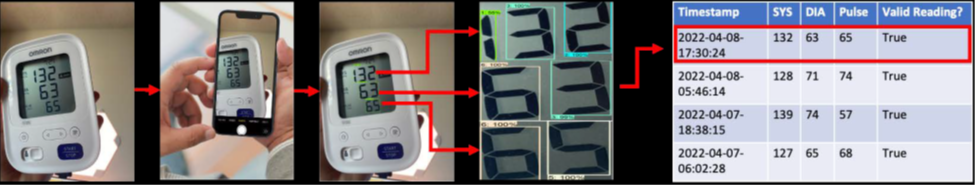
**

Deep Learning

There were three problems that we needed to solve: measure detection (finding the blood pressure cuff in the image); digit detection (finding the digits in the image); digit classification (determining which digits were in the image). These three problems were to be solved using two different models: one for measure detection and one for digit detection and classification. The TensorFlow Object Detection API(1) was utilized to create and train these models, and each used a transfer learning approach based on the present weights. During the process, several model architectures were tested to determine which would be best suited for the tasks at hand.

For all models trained, the COCO detection metrics (2) were used to evaluate the model during the training process. These metrics utilize precision and recall values at different object scales. For each model, a different metric was used as a primary means of evaluating its performance based on its primary function.

Training Model 1
The first model developed to detect the measures in the images was based on the EfficientDet D1 640x640 model from the TensorFlow Model Zoo.(1) The main metric of interest for this model was the localization loss as this model’s primary purpose was to find the three different measures within the given image.

Training Model 2

The final iteration of the second model developed to detect and classify the digits in the image was also based on the EfficientDet D1 model from the Model Zoo.(1) This model was trained for 10,000 steps until the classification and localization loss converged.

Evaluation and Feedback Pipeline

To classify new images, the two trained models were combined. The three cropped outputs of the measure detection model were fed into the digit detection model to get the blood pressure values. The final step of the process was to define a rejection metric that would reject unreasonable values. An image was accepted if 3 measurements were detected in the image; systolic BP was between 50 and 300; diastolic BP was between 20 and 200; systolic BP larger than diastolic BP; and pulse was between 30 and 200.

Appendix References

1. Abadi M, Barham P, Chen J, Chen Z, Davis A, Dean J, et al., editors. {TensorFlow}: a system for {Large-Scale} machine learning. 12th USENIX symposium on operating systems design and implementation (OSDI 16); 2016.

2. Lin T-Y, Maire M, Belongie S, Hays J, Perona P, Ramanan D, et al., editors. Microsoft coco: Common objects in context. Computer Vision–ECCV 2014: 13th European Conference, Zurich, Switzerland, September 6-12, 2014, Proceedings, Part V 13; 2014: Springer.
